# Structure and physicochemical characterization of a naproxen–picolinamide cocrystal

**DOI:** 10.1107/S2053229616011980

**Published:** 2017-02-06

**Authors:** Hannah E. Kerr, Lorna K. Softley, Kuthuru Suresh, Paul Hodgkinson, Ivana Radosavljevic Evans

**Affiliations:** a Department of Chemistry, Durham University, Lower Mountjoy, Stockton Road, Durham DH1 3LE, UK; b School of Chemistry, University of Hyderabad, Hyderabad 500 046, India

**Keywords:** naproxen, picolinamide, cocrystal, NMR crystallography, DFT analysis, hydrogen bonding, crystal structure, pharmaceuticals, dimers, computational chemistry

## Abstract

Naproxen (NPX) is a nonsteroidal anti-inflammatory drug with pain- and fever-relieving properties, currently marketed in the sodium salt form to overcome solubility problems; however, alternative solutions for improving its solubility across all pH values are desirable. NPX is suitable for cocrystal formation, with hydrogen-bonding possibilities *via* the COOH group. The crystal structure is presented of a 1:1 cocrystal of NPX with picolinamide as a coformer [systematic name: (*S*)-2-(6-meth­oxy­naphthalen-2-yl)propanoic acid–pyridine-2-carbox­amide (1/1), C_14_H_14_O_3_·C_6_H_6_N_2_O]. The pharmaceutically relevant physical properties were investigated and the intrinsic dissolution rate was found to be essentially the same as that of commercial naproxen. An NMR crystallography approach was used to investigate the H-atom positions in the two crystallographically unique COOH–CONH hydrogen-bonded dimers. ^1^H solid-state NMR distinguished the two carboxyl protons, despite the very similar crystallographic environments. The nature of the hydrogen bonding was confirmed by solid-state NMR and density functional theory calculations.

## Abstract 2

The crystal structure is reported of a new 1:1 cocrystal of naproxen with picolinamide, and the pharmaceutically relevant properties are investigated. An NMR crystallography approach is used to distinguish between two crystallographically unique COOH–CONH hydrogen-bonded dimers and to confirm the location of the H atoms in the two dimers.

## Introduction   

Naproxen (NPX, Scheme 1), or (*S*)-2-(6-meth­oxy­naphthalen-2-yl)propanoic acid, is a nonsteroidal anti-inflammatory drug with pain- and fever-relieving properties, commonly used in the treatment of arthritis, dysmenorrhea and acute gout. NPX is a weak acid (*DrugBank*, accessed July 2016; Wishart *et al.*, 2006 ▸), with a p*K_a_* value of 4.2, leaving it un-ionized in gastrointestinal fluids. It is formally classified as a low-solubility high-permeability drug (Takagi *et al.*, 2006 ▸), but its solubility is highly dependent on the pH of the surrounding environment. The large hydro­phobic aromatic region present in the mol­ecule disfavours inter­actions with water mol­ecules, and hence it is insoluble in aqueous media. In environments with higher pH, such as in the membranes surrounding the cells, NPX becomes ionized, with the charged COO^−^ group forming more favourable inter­actions with water, enhancing dissolution. To overcome the solubility problems, NPX is currently marketed in a salt form, *i.e.* naproxen sodium; however, alternative solutions for improving the solubility of NPX across all pH values are desirable.

**Figure 1:**
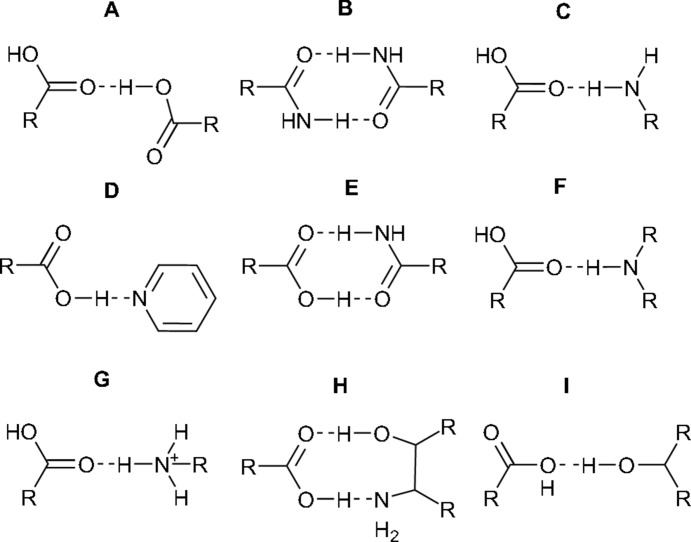
Synthons present in the NPX cocrystals previously reported in the literature. Note that synthon **B** involves neighbouring INA mol­ecules, and the NPX COOH group is involved in synthons **C** and **D**.

NPX is suitable for cocrystal formation, with hydrogen-bonding possibilities *via* the COOH group, which can form a range of one-, two- or three-dimensional robust synthons (Desiraju, 1995 ▸), with additional π-stacking inter­actions from the aromatic region. To date, 16 cocrystals of NPX have been synthesized and characterized using a variety of techniques, including X-ray diffraction (XRD), infrared (IR) spectroscopy and differential scanning calorimetry (DSC). Cocrystals with the following coformers have been reported: nicotinamide (NA), isonicotinamide (INA), picolinamide (PA) (Neurohr *et al.*, 2015 ▸; Castro *et al.*, 2011 ▸), *trans*-1,2-bis­(pyridin-4-yl)ethyl­ene (TBPE) (Weyna *et al.*, 2009 ▸), duloxetine (Buschmann *et al.*, 2009 ▸), tramadol (Buschmann *et al.*, 2010 ▸), bi­pyridine (BPY) and piperazine (PPZ) (Manoj *et al.*, 2014 ▸), and several chiral amino acids, including alanine (AL), zwitterionic prolinium (PR), tyrosine (TY), tryptophan (TP) and *N*-octylglucamine (O-GL) (Tumanova *et al.*, 2014 ▸; Tilborg *et al.*, 2013 ▸; Yuan *et al.*, 2001 ▸). The structures of many of these cocrystals have been determined using single-crystal diffraction (see footnote in Table 1 ▸). The relevant synthons are shown in Fig. 1 ▸ and summarized for all 16 reported cocrystals in Table 1 ▸.
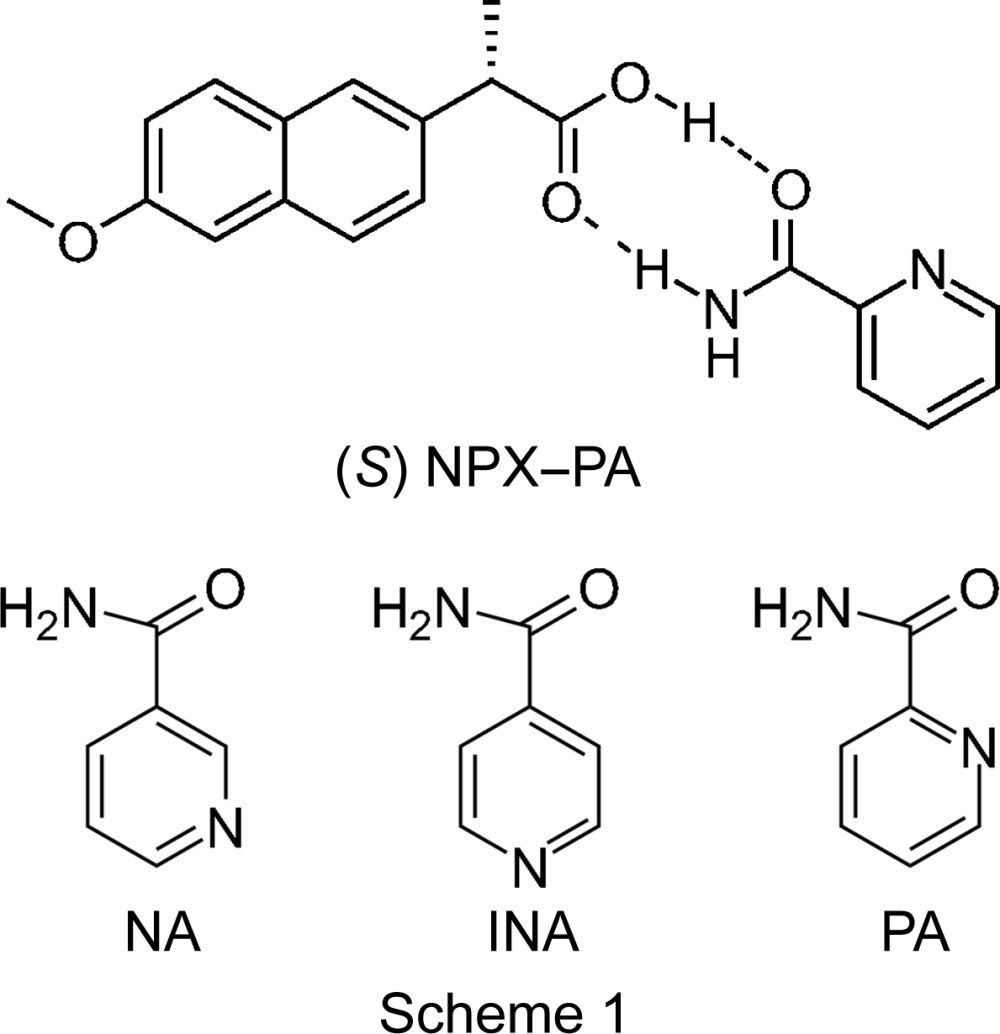


The coformers of five of the reported cocrystals contain a pyridine ring, which readily inter­acts with the carb­oxy­lic acid group on NPX (synthon **D**). The importance of synthon **D** is further highlighted by observing the synthons present in cocrystals with the isomeric pyridinecarboxamides NA and INA coformers (see Scheme 1). The formation of synthon **D** in 2NPX–NA prevents the formation of synthon **B** (seen in NPX–INA), due to the unfavourable distortion required for the groups in the correct orientation to inter­act (Ando *et al.*, 2012 ▸). Consequently, synthon **E** is formed instead. Synthon **A** is present in most of the NPX–amino acid cocrystals, highlighting the preference for the formation of carb­oxy­lic acid–carb­oxy­lic acid dimers. The preparation of cocrystals between NPX and the third pyridine carboxamide isomer, picolinamide (PA, Scheme 1), has been suggested by Castro *et al.* (2011 ▸); however, single crystals were not available and so no X-ray structure was determined.

Solid–state NMR (SS–NMR) is sensitive to the local structure, and NMR crystallography studies have been employed extensively to characterize hydrogen bonding in a variety of cocrystals (Harris *et al.*, 2009 ▸; Stevens *et al.*, 2014 ▸; Vogt *et al.*, 2009 ▸; Gobetto *et al.*, 2005 ▸; Chierotti & Gobetto, 2013 ▸; Brown, 2012 ▸), including cocrystals of NPX. For example, 2NPX–NA was investigated by two-dimensional (2D) ^1^H–^13^C heteronuclear correlation (HETCOR) experiments that confirmed the hydrogen-bonding network was a relatively rare example of a single carboxyl group giving rise to two different inter­molecular synthons in the same cocrystal, probably due to the 2:1 stoichiometry (Ando *et al.*, 2012 ▸). Pure NPX and its sodium salt have also been studied by SS–NMR methods, with full chemical-shift assignment and ring-current effects investigated in 2013 (Carignani *et al.*, 2013 ▸), while ^23^Na SS–NMR experiments were also used to study NPX hydrates and solvates (Burgess *et al.*, 2012 ▸). The ^13^C and ^1^H spectral assignments of NPX have been critically analysed recently using calculated NMR parameters with density functional theory (DFT), with three correlations from the paper by Ando *et al.* (2012 ▸) reassigned in the crowded region of the ^1^H–^13^C HETCOR spectra (Czernek, 2015 ▸). The tetra­hydrate sodium salt of NPX has a complex disordered sodium and water network, which can be inter­preted in terms of either stacking faults or multiple twinning following an NMR crystallography study utilizing SS–NMR, XRD and computational methods (Bond *et al.*, 2013 ▸).

SS–NMR is well suited to the study of hydrogen-bonding networks in cocrystals (Maruyoshi *et al.*, 2012 ▸; Tatton *et al.*, 2013 ▸; Dudenko *et al.*, 2013 ▸; Reddy *et al.*, 2015 ▸), including locating protons in short hydrogen bonds. A combination of ^1^H and ^15^N SS–NMR is commonly used to determine proton positions over O—H⋯O and O—H⋯N hydrogen bonds due to the sensitivity of the chemical shift to the local environment. For example, two sulfa­thia­zole–oxalic acid complexes have been shown to be salts by multinuclear SS–NMR (Koike *et al.*, 2014 ▸) and recently a furosemide–isonicotinamide complex was confirmed to be a cocrystal by comparison of ^15^N SS–NMR and DFT-calculated chemical shifts (Kerr *et al.*, 2015 ▸). Another aspect of the study of proton position is the characterization of reversible proton migration over short strong hydrogen bonds, which is of fundamental inter­est but also potentially relevant to some types of functional materials (Ford *et al.*, 2011 ▸; Frantsuzov *et al.*, 2014 ▸).

We report here the growth of NPX–PA single crystals, the structure determination by single-crystal X-ray diffraction, the preparation of a pure polycrystalline sample of this cocrystal and the determination of its pharmaceutically-relevant properties. The details of the hydrogen-bonding network are investigated with NMR crystallography methods.

## Experimental   

### Synthesis and crystal growth   

NPX–PA was synthesized *via* a mechanochemical route. Equimolar (0.5 mmol) amounts of (*S*)-NPX and PA were ground together using a mortar and pestle for 30 min with the gradual addition of a total of 6 drops of ethanol. A soft white powder was produced. The sample was reground for 30 min with the addition of a further 4 drops of ethanol in order to obtain the cocrystal as a single polycrystalline phase.

A small portion (3 mg) of the polycrystalline material was refluxed in acetone at approximately 353 K for 20 min. Solutions were left to evaporate in vials with pierced lids both at room temperature and in a fridge. Clear rectangular plate-like crystals appeared in both vials after 24 h.

### Single-crystal X-ray diffraction   

Crystal data, data collection and structure refinement details are summarized in Table 2 ▸. Crystals that had crystallized at both temperatures (§2.1) were screened. They were the same product and the crystals were of similar quality. H atoms not involved in hydrogen bonding were placed geometrically and treated using a riding model (Cooper *et al.*, 2010 ▸). The six H atoms potentially involved in hydrogen bonding (H31, H201, H202, H291, H292 and H361) were located from difference Fourier maps, and their fractional coordinates and isotropic atomic displacement parameters were freely refined. The Flack (1983 ▸) parameter could not be determined reliably.

### Powder X-ray diffraction   

Powder X-ray diffraction (PXRD) data were collected on a Bruker D8 ADVANCE diffractometer (Cu *K*α_1,2_ radiation) and a LynxEye detector. Patterns were recorded in ranges between 4 and 50° using a step size of 0.014°. Data analysis was carried out by the Rietveld method (Rietveld, 1969 ▸) using *TOPAS-Academic* software (Coelho *et al.*, 2011 ▸).

### Solid-state nuclear magnetic resonance (SS–NMR)   

^13^C cross-polarization (CP)/magic-angle spinning (MAS) measurements of pure NPX, a physical mix of NPX and PA, and the NPX–PA cocrystal were recorded on a Varian VNMRS 400 spectrometer using an 8 kHz spinning rate, a recycle delay of 5 s and a contact time of 5.0 ms. TPPM decoupling was applied during acquisition with a ^1^H nutation rate of 74 kHz. Spectra were referenced to neat TMS by setting the high shift resonance of a replacement sample of adamantane to 38.5 ppm.

All other spectra were recorded on a Bruker Avance III HD spectrometer operating at a ^1^H frequency of 499.6 MHz and a ^13^C frequency of 125.7 MHz. The ^1^H–^13^C FSLG–HETCOR experiment was carried out at 10 kHz MAS with a 20 s recycle delay and a 1 ms contact time. 64 *t*_1_ increments were acquired with 48 transients per increment. ^13^C spectra were referenced by setting the carbonyl resonance of a replacement sample of α-glycine to 176.5 ppm. The ^1^H experiment was carried out on a 1.3 mm probe (rotor outer diameter) at a spinning rate of 60 kHz with a recycle delay of 20 s. The ^1^H spectrum was referenced by setting the resonance of a replacement sample of adamantane to 1.9 ppm. The ^1^H dimensions of the HETCOR spectra were rescaled using the default FSLG scaling factor and then referenced using resolved peaks from the ^1^H MAS spectrum.

### Computational methods   

First principles calculations were carried out using the GIPAW method implemented in *CASTEP* (Clark *et al.*, 2005 ▸). All calculations were performed using the PBE functional (Perdew *et al.*, 1996 ▸) and on-the-fly-generated ultrasoft pseudo­potentials, with a cut-off energy of 700 eV. Geometry optimization of all 184 atom positions was carried out with the centre of mass and unit-cell parameters fixed, with integrals taken over the Brillouin zone using a Monkhorst–Pack grid with a maximum *k*-point sample spacing of 0.1 Å^−1^, corresponding to a single *k*-point. As discussed below, alternative hydrogen-bonding models were produced by moving the H atoms along the vector of the hydrogen bonds in 16 equal increments by editing the Cartesian coordinates of the geometry-optimized structure. The energy was calculated at each position using a single-point calculation, with a cut-off energy of 700 eV. Geometry optimization including dispersion correction did not significantly change the H-atom positions or predicted shielding values.

The NMR parameters were calculated using a *k*-point sample spacing of 0.05 Å^−1^ corresponding to 4 *k*-points and an offset of (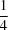, 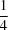, 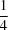) to avoid sampling the Γ point. The resulting shielding values were converted to chemical shifts and referenced using δ_iso_ = σ_ref_ − σ_iso_, where σ_iso_ is the *CASTEP*-calculated shielding value and σ_ref_ was calculated to equate the average calculated shift and average experimental shift (Harris *et al.*, 2007 ▸). The σ_ref_ values are given in Table S3 in the *Supporting information*, along with the r.m.s. deviations (RMSDs) of the calculated ^1^H and ^13^C NMR parameters compared for the different hydrogen-bonding models.

### Thermal analysis   

Differential scanning calorimetry (DSC) measurements were carried out using a TA DSC Q1000 instrument equipped with a nitro­gen purge gas, using 4–5 mg of sample. The heating rate was 10 K min^−1^.

### Property measurements   

To compare the solubility of NPX and the NPX–PA cocrystal, intrinsic dissolution rate (IDR) and equilibrium solubility studies in pH 7 phosphate buffer medium were performed. The equilibrium solubility of NPX was measured (4.32 g l^−1^), but that of the NPX–PA cocrystal could not be determined since the material dissociated to its starting components (as observed by PXRD). The IDR experiments were carried out on a USP-certified Electrolab TDT-08L dissolution tester type II (paddle) (Mumbai, India) for 6 h (Higuchi & Connors, 1965 ▸). Prior to IDR determination, standard curves for both NPX and NPX–NA were obtained spectrophotometrically.

## Results and discussion   

### Single-crystal X-ray diffraction and physicochemical properties   

**Figure 2:**
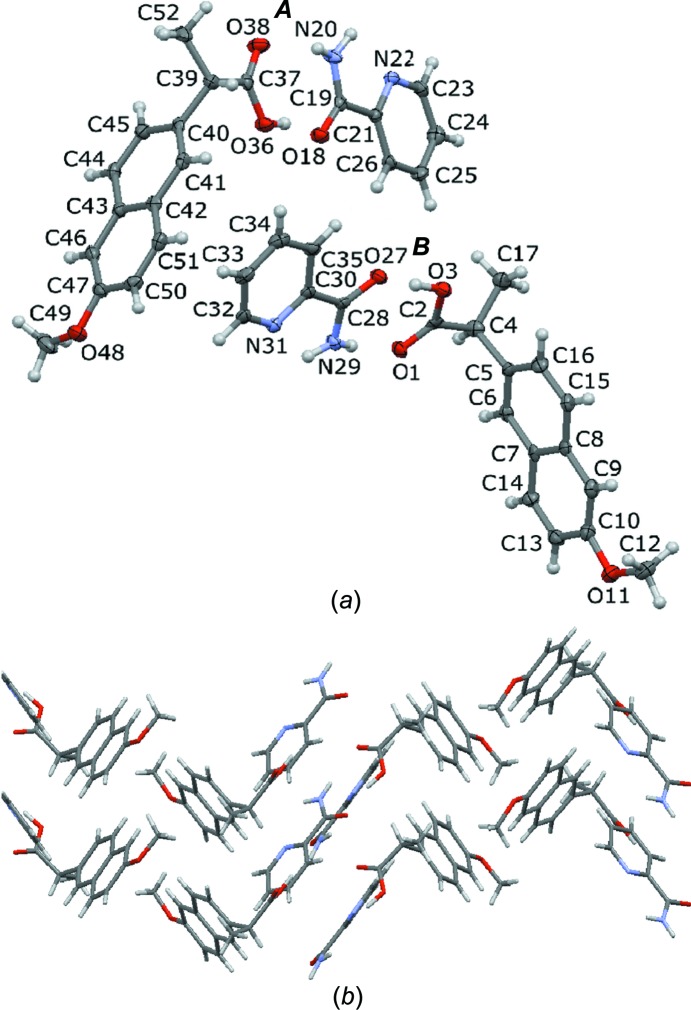
(*a*) The asymmetric unit of NPX–PA, with displacement ellipsoids shown at the 50% probability level and heavy atoms labelled. H atoms are shown with a fixed radius of 0.15 Å. (*b*) The crystal packing in NPX–PA, viewed along the *c* axis.

NPX–PA crystallizes in the space group *P*2_1_ with four mol­ecules in the asymmetric unit [two active pharmaceutical ingredient (API) and two conformer mol­ecules]. The mol­ecules form heterodimers *via* synthon **E** (Fig. 1 ▸). The presence of four mol­ecules in the asymmetric unit yields two inequivalent **E** heterodimers, arranged in an edge-to-face herringbone pattern along the crystallographic *c* axis. The dimers inter­act with each other *via* synthon **C**, with an O⋯N distance of 3.022 (4) Å. The synthons and their packing can be seen in Fig. 2 ▸ and details of the hydrogen-bonding distances in NPX–PA are summarized in Table 3 ▸. These distances fall within the range observed in other structures containing the synthon **E** dimer (see Fig. S1 in the *Supporting information*). The structural model obtained from single-crystal diffraction was used to fit the PXRD data obtained on the bulk sample. The Rietveld fit obtained is shown in Fig. S3 (see *Supporting information*) and no peaks are unaccounted for, implying that the polycrystalline material prepared by mechanochemistry is a single-phase product.

The structure obtained is consistent with the trends seen in previously determined NPX cocrystal structures. In particular, the heterodimer formation between carb­oxy­lic acid and amide groups, linked by a further single inter­action is reminiscent of the synthon formation in 2NPX–NA and NPX–INA. However, synthon **D** is not observed in NPX–PA, despite being present in 2NPX–NA and NPX–INA, and alternatively synthon **E** is formed. This demonstrates the role of basicity in cocrystal formation with NPX. Among the three pyridine­carboxamide coformers, NA and INA are more basic (p*K_a_* values of 3.63 and 3.45), so the acid–pyridine synthon **D** is observed. Additionally, in 2NPX–NA, the other NPX mol­ecule is associated with NA *via* the acid–amide synthon **E**. However, PA exhibits the *ortho* effect and is less basic [p*K*_a_ value of 1.17 (Mihala, 2016 ▸; ChemAxon, 2016 ▸)], so the PA carboxamide group inter­acts with NPX *via* synthon **E** (acid–amide) rather than synthon **D**.

**Figure 3:**
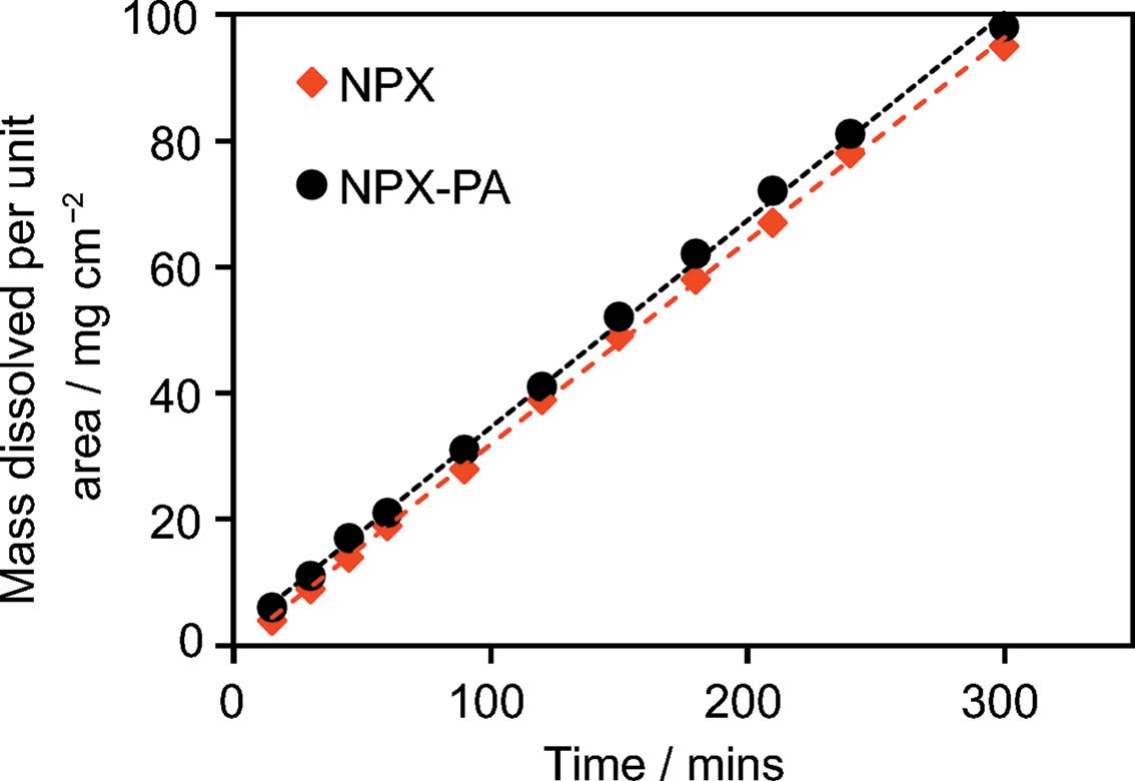
Dissolution curves for pure NPX (black squares) and the NPX–PA cocrystal (red circles) measured over a 6 h period.

The DSC trace of NPX–PA shows a single endothermic peak at 366 K, which represents the melting of a single solid phase (see Fig. S4 in the *Supporting information*). This is in keeping with the endothermic peak observed at 364 K by Castro *et al.* (2011 ▸), and is lower than that of both starting components (NPX 425 K and PA 375 K). The intrinsic dissolution rates of NPX and the NPX–PA cocrystal were found to be 1.26 and 1.39 mg cm^−2^ min^−1^, respectively, *i.e.* the NPX–PA cocrystal dissolves at essentially the same rate as commercial NPX (Fig. 3 ▸). The identity of the undissolved materials at the end of the dissolution experiment was confirmed to be unchanged by PXRD.

One question that arises from the structure determination concerns the O—H⋯O hydrogen bonds in the two crystallographically unique synthon **E** dimers. While the donor–acceptor (*D*–*A*) distances are essentially the same (see Table 3 ▸), the situation with the donor–hydrogen (*D*—H) bond lengths is less clear-cut given the larger standard uncertainties of these parameters and systematic issues with locating H-atom positions using X-ray scattering. In particular, the *D*—H distances might suggest that the crystallographically unique COOH–CONH dimers are significantly different, but the large standard errors of the H-atom positions preclude any definitive conclusions. Given the strong sensitivity of NMR shifts to the local structure, it is expected that NMR spectra would help to confirm the location of the H atoms within the hydrogen bonds.

### NMR crystallography   

**Figure 4:**
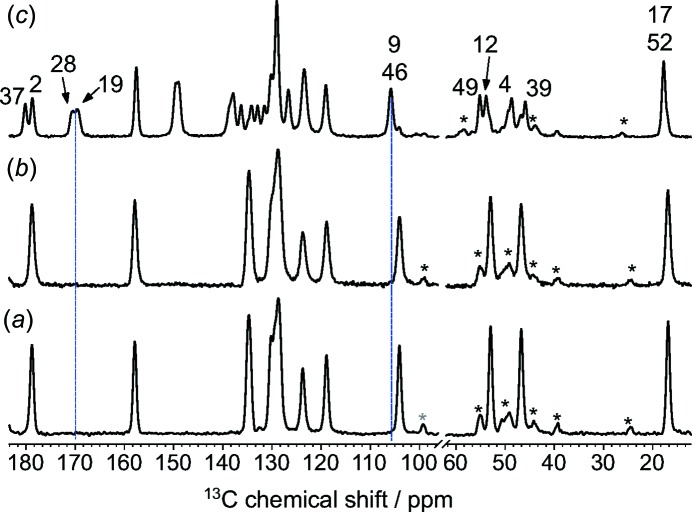
^13^C CP/MAS spectra of (*a*) pure NPX, (*b*) a physical mixture of NPX and PA, and (*c*) the NPX–PA cocrystal acquired at a ^13^C frequency of 100.56 MHz. Peaks marked with an asterix are spinning sidebands and the dashed blue lines are guides for the eye to show new or shifted peaks in the cocrystal.

**Figure 5:**
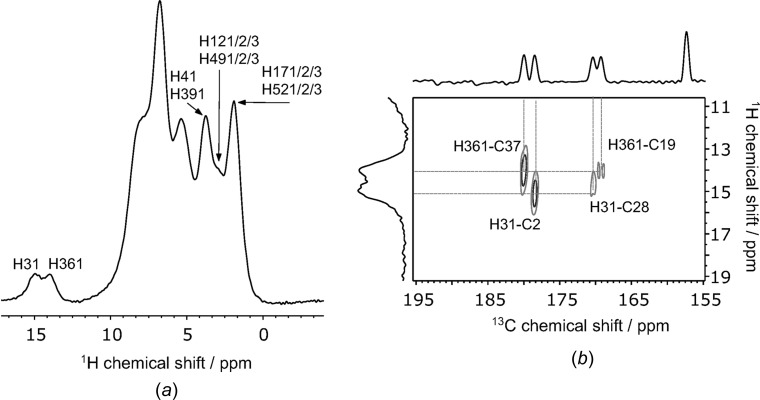
(*a*) The ^1^H NMR spectrum of NPX–PA at 60 kHz MAS. Atom labelling is given for some sites such that the H-atom label is H*X*1, where *X* is the label of the directly bonded heavy atom, for example, the H atom on O36 is H361. (*b*) A section of the ^1^H–^13^C HETCOR spectrum of NPX–PA, with the hydrogen-bonded proton peaks and the NPX carboxyl carbon peaks labelled.

Comparison of the ^13^C spectra of the pure components with that of the grinding product confirms the formation of a cocrystal (Fig. 4 ▸). The physical mixture and pure NPX show identical spectra, consistent with a lack of inter­action between components. Peaks from PA are not observed because the pure PA coformer has a long ^1^H *T*_1_ relaxation time compared to the recycle delay of 5 s. The spectrum of the NPX–PA cocrystal is distinctly different. Firstly, some of the resonances arising from NPX have shifted relative to the pure NPX spectrum, *e.g.* the C9/C46 peak. The deshielding observed for the NPX COOH sites, *i.e.* C2 and C37, is consistent with the formation of hydrogen bonds (Asakawa *et al.*, 1992 ▸). Secondly, signals are now observed from PA carbon sites as a result of the intimate association of PA and NPX, which shortens the ^1^H *T*_1_ relaxation time of the PA resonances. The doubling of most signals is consistent with the presence of two crystallographically non-equivalent mol­ecules of NPX and PA in the asymmetric unit determined from single-crystal X-ray diffraction. Key peaks are assigned in Fig. 5 ▸, and the full assignment is given in Fig. S5 and Table S3 in the *Supporting information*. The spectral assignments were aided by a ^13^C spectrum with the nonquaternary carbon peaks suppressed (Fig. S4 in the *Supporting information*) and 2D ^1^H–^13^C heteronuclear correlation (HETCOR) experiments (Figs. S5 and S6 in the *Supporting information*), as well as *CASTEP*-calculated shieldings (Figs. S8 and S9 in the *Supporting information*), discussed below. A direct-excitation ^13^C experiment (not shown) with a short recycle delay of 0.5 s showed that the only obviously dynamic sites are the methyl groups.

**Figure 6:**
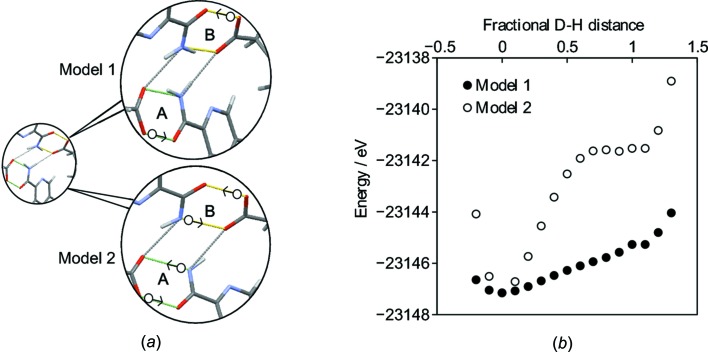
(*a*) Schematic showing the H atoms that were moved in tandem along the vector between the H atom and its acceptor atom to form Model 1 and Model 2. The white circles indicate the initial position of the H atoms from the XRD structure and the arrows indicate the direction of movement. (*b*) The energy of NPX–PA as a function of H-atom position. A fractional distance of 0 corresponds to the DFT-optimized XRD position, while a distance of 1 corresponds to the H atom being at the equivalent position on the other side of the hydrogen bond. Negative fractional distances correspond to movement towards the donor atom.

The ^1^H spectrum of NPX–PA acquired with fast MAS of 60 kHz (Fig. 5 ▸ *a*), shows comparable resolution with that of the 2NPX–NA spectrum presented by Ando *et al.* (2012 ▸). It is likely that the chemical shifts of some of the protons are affected by inter­molecular ring-current effects due to the edge-to-face herringbone structure of NPX–PA, which is similar to that of pure NPX (Carignani *et al.*, 2013 ▸). Whilst the broad linewidths of ^1^H SS–NMR can hinder the ability of SS–NMR to distinguish between protons in hydrogen bonds of the same type, there are many cocrystals/salts for which it is possible (Vogt *et al.*, 2009 ▸; Gobetto *et al.*, 2005 ▸; Sardo *et al.*, 2015 ▸; Harris *et al.*, 2010 ▸), including this case of NPX–PA. Not all ^1^H peaks could be assigned, but the peaks of the two protons involved in the synthon **E** dimers, *i.e.* H31 and H361, can be distinguished. Variable recycle delay experiments showed that the *T*_1_ relaxation of the hydrogen-bonded protons was significantly slower than that of other H atoms, and so a 20 s recycle delay was used to acquire the spectra shown in Fig. 6 ▸.

Atoms H31 and H361 can be assigned unambiguously due to the observation of strong correlations to the NPX carbonyl C atoms (C2/C37) in a HETCOR spectrum acquired with a 1 ms contact time (Fig. 6 ▸ *b*). Additionally, weak correlations are observed to C19/C28, the PA amide C atoms involved in the dimers of synthon **E** (H31⋯C28 = 2.41 Å and H361⋯C19 = 2.39 Å). Similarly, a ^1^H–^1^H double quantum/single quantum spectrum (DQ/SQ) (see Fig. S9 in the *Supporting information*), also shows direct evidence of the synthon **E** dimers by means of correlations between H31/H361 and the amide protons H291/H202. The HETCOR spectra also help to confirm the positioning of the H atoms; the C19/C28 correlations would be expected to be more intense than the C2/C37 correlations if the protons were nearer the amide C atoms in the N—H⋯O hydrogen bond. The observed correlation intensities, therefore, support the assignment of hydrogen-bond donors and acceptors by single-crystal XRD.

The positions of atoms H31 and H361 within the hydrogen bonds is further investigated by comparing the experimental NMR data to predicted shift values. The structure obtained from XRD was first geometry optimized using DFT. Optimization of all the atom positions results in a structure with a small total heavy-atom RMSD between the optimized and original structures of 0.09 Å. As would be expected, however, the positions of H atoms refined from X-ray scattering are significantly adjusted, by ∼0.2 Å, on optimization. This is within the usual range observed when comparing hydrogen-bond distances from X-ray scattering and neutron scattering experiments (Wells, 1984 ▸). As shown in Table S1 (see *Supporting information*), the hydrogen bonds become more symmetric and the difference between the two O—H⋯O hydrogen bonds largely disappears, which is consistent with the small difference (∼1 p.p.m.) in the ^1^H shifts for atoms H31 and H361.

Two hydrogen-bonding models can be proposed as alternatives to the XRD refinement, see Fig. S9(i) in the *Supporting information*. In Model 1, only the H atoms in the short O—H⋯O hydrogen bonds were moved, and in Model 2 the amide protons in PA were also moved along the N—H⋯O hydrogen bond. Specifically, atom H361 was moved from O36 to O18 and atom H31 was moved from O3 to O27 in dimers *A* and *B*, respectively, in Model 1. In Model 2, atom H202 was also moved from N20 to O38 and atom H291 was moved from N29 to O1 in dimers *A* and *B*, respectively. Note that there is a net proton transfer in Model 1, resulting in a salt form, while Model 2 is an alternative cocrystal (see Fig. S2 in the *Supporting information*). The calculated potential energy as a function of H-atom position for these models is shown in Fig. 6 ▸(*b*). There is a single energy minimum in both cases, corresponding to the structure determined from XRD. It is important to note that DFT calculations have some systematic weaknesses in describing hydrogen bonding, *e.g.* in a furosemide–isonicotinamide cocrystal, the energetic minimum in a short strong O—H⋯N hydrogen bond was predicted incorrectly, as shown by experimental ^15^N NMR and XRD data (Kerr *et al.*, 2015 ▸). Moreover, the DFT calculations effectively involve 0 K structures, while experimental SC–XRD and NMR were carried out at 120 K and ambient temperature, respectively. However, the potential curves in Fig. 6 ▸(*b*) each show a single steep minimum, so alternative positions for the H atoms are implausible and temperature-dependent proton transfer over the hydrogen bonds is highly unlikely. This contrasts with a previously reported system, namely pyridine-3,5-di­carb­oxy­lic acid, where an NMR crystallography study revealed the presence of quantum tunnelling of a proton across a hydrogen bond with a shallow double minimum in the potential energy surface (Frantsuzov *et al.*, 2014 ▸). In this context, it is important to note that only the NMR parameters calculated from the geometry-optimized XRD structure are compatible with the experimental data, as discussed in Figs. S8 and S9 in the *Supporting information*. In particular, the RMSDs between experimental and calculated ^13^C shifts are over 2 p.p.m. (Table S2 in the *Supporting information*) if the H atoms are moved to the other side of the hydrogen bond, according to either Model 1 or Model 2, which is inconsistent with valid structures for mol­ecular organics (Bonhomme *et al.*, 2012 ▸; Widdifield *et al.*, 2016 ▸).

## Conclusions   

The crystal structure of NPX–PA was refined from single-crystal XRD and was found to contain two crystallographically unique NPX and PA mol­ecules that form two carb­oxy­lic acid–carboxamide dimers between NPX and PA. The intrinsic dissolution rate of NPX–PA was found to be the same as that of commercial NPX and the apparent solubility could not be measured because the cocrystal dissociated into the pure starting components. This contrasts with previously described cocrystals of NPX, which have improved dissolution rates compared to pure NPX and low hygroscopicity.

DFT geometry optimization was used to refine the positions of the H atoms, resulting in an excellent agreement with experimental evidence from ^13^C and ^1^H solid-state NMR. The calculated potential energies as a function of H-atom position validate the overall positioning determined by XRD, confirming that NPX–PA is a cocrystal and not a salt. The hydrogen-bonded H atoms in the two crystallographically unique dimers could be distinguished experimentally, despite the two environments being very similar in the geometry-optimized structures, demonstrating the sensitivity of NMR spectra to H-atom position.

## Supplementary Material

Crystal structure: contains datablock(s) I. DOI: 10.1107/S2053229616011980/df3001sup1.cif

Structure factors: contains datablock(s) I. DOI: 10.1107/S2053229616011980/df3001Isup2.hkl

H-bond dimensions in geometry optimised structure; NMR reference data; bond length distributions in COOH-CONH dimers; PXRD and DSC results; detailed information on NMR assignment. DOI: 10.1107/S2053229616011980/df3001sup3.pdf

CCDC reference: 1495366

## Figures and Tables

**Table 1:** Synthons present in previously reported NPX cocrystals NA is nicotinamide, INA is isonicotinamide, TBPE is *trans*-1,2-bis­(pyridin-4-yl)ethyl­ene, BPY is bi­pyridine, PPZ is piperazine, AL is alanine, TY is tyrosine, PR is zwitterionic prolinium, TP is tryptophan and O-GL is *N*-octylglucamine.

	**A**	**B**	**C**	**D**	**E**	**F**	**G**	**H**	**I**
*S*-NPX^*a*^	*								
*S*-2NPX–NA^*b*^			*	*	*				
*S*-NPX–INA^*b*^		*	*	*					
*S*-NPX–TBPE^*c*^				*					
*RS*-NPX–BPY^*d*^				*					
*S*-NPX–BPY^*d*^				*					
*RS*-NPX–PPZ^*d*^						*			
*S*-NPX–PPZ^*d*^						*			
*S*-NPX–L-AL^*e*^	*						*		
*S*-NPX–D-AL^*e*^	*						*		
*S*-NPX–D-TY^*e*^	*						*		*
*S*-NPX–D-TP^*e*^	*					*	*		
*S*-NPX–L-PR^*e*^	*		*						
*S*-NPX–D-PR^*e*^	*		*						
*RS*-NPX–L-PR^*e*^	*								
*RS*-NPX–DL-PR^*e*^	*		*						
*S*-NPX–O-D-GL^*e*^						*		*	*

References: (*a*) Ravikumar *et al.* (1985 ▸); (*b*) Neurohr *et al.* (2015 ▸) and Castro *et al.* (2011 ▸); (*c*) Weyna *et al.* (2009 ▸); (*d*) Manoj *et al.* (2014 ▸); (*e*) Tumanova *et al.* (2014 ▸), Tilborg *et al.* (2013 ▸) and Yuan *et al.* (2001 ▸).

**Table 2:** Experimental details

Crystal data	
Chemical formula	C_14_H_14_O_3_·C_6_H_6_N_2_O
*M*_r_	352.39
Crystal system, space group	Monoclinic, *P*2_1_
Temperature / K	120
*a*, *b*, *c* / Å	5.3048 (5), 31.891 (3), 10.508 (1)
β / °	98.184 (3)
*V* / Å^3^	1759.6 (3)
*Z*	4
Radiation type	Mo *K*α
μ / mm^−1^	0.09
Crystal size / mm	0.12 × 0.04 × 0.02

Data collection	
Diffractometer	Bruker Venture D8
No. of measured, independent and observed [*I* > 2σ(*I*)] reflections	19879, 8939, 5437
*R*_int_	0.045
(sin θ/λ)_max_ / Å^−1^	0.717

Refinement	
*R*[*F* ^2^ > 2σ(*F* ^2^)], *wR*(*F* ^2^), *S*	0.058, 0.082, 0.94
No. of reflections	5437
No. of parameters	493
No. of restraints	1
H-atom treatment	H atoms treated by a mixture of independent and constrained refinement
Δρ_max_, Δρ_min_ / e Å^−3^	0.39, −0.32

Computer programs: *APEX2* (Bruker, 2012 ▸), *SIR92* (Altomare *et al.*, 1993 ▸) and *CRYSTALS* (Betteridge *et al.*, 2003 ▸).

**Table 3:** The geometry (Å, °) of the hydrogen bonds in the NPX–PA single-crystal XRD structure

Dimer	Hydrogen bond	*D*—H	H⋯*A*	*D*⋯*A*	*D*—H⋯*A*
*A*	O36—H361⋯O18	0.85 (4)	1.74 (4)	2.572 (5)	166 (4)
	N20—H202⋯O38	0.85 (4)	2.15 (4)	2.965 (5)	158 (4)
*B*	O3—H31⋯O27^i^	0.97 (6)	1.62 (6)	2.579 (5)	169 (6)
	N29—H291⋯O1^ii^	0.81 (4)	2.08 (4)	2.890 (5)	175 (4)

Symmetry codes: (i) *x* − 1, *y*, *z*; (ii) *x* + 1, *y*, *z*.
