# Supplementary material for: Structure and physicochemical characterization of a naproxen–picolinamide cocrystal
Source: Acta Crystallogr C Struct Chem. 2017 Feb 6;73(Pt 3):168–75. doi: 10.1107/S2053229616011980 (PMC5391861; doi:10.1107/S2053229616011980)
Supplement: Supplementary file 3 [file c-73-00168-sup3.pdf]

Supporting information

Table S1 Hydrogen bond dimensions in geometry-optimised NPX-PA.

Dimer	Hydrogen Bond	$d_{X—H}$ / Å	$d_{H\cdots A}$ / Å	$d_{X\cdots A}$ / Å	Bond angle / °
A	O36—H361···O18	1.05	1.49	2.534	169
	N20—H202···O38	1.03	1.89	2.897	166
B	O3—H31···O27	1.05	1.51	2.546	170
	N29—H291···O1	1.03	1.83	2.844	167

Table S2 σ_{ref} values used to reference the simulated NPX-PA ^{13}C and ^{1}H spectra and RMSD differences between calculated and experimental ^{13}C shifts.

Structure	^{1}H σ_{ref} / ppm	^{13}C σ_{ref} / ppm	^{13}C RMSD / ppm
XRD-refined	31	170	1.6
Model 2	30	171	2.7
Model 1	30	169	2.4

σ_{ref} values obtained using $\delta_{iso} = \sigma_{ref} - \sigma_{iso}$, where σ_{iso} is the CASTEP-calculated shielding value and σ_{ref} was calculated to equate the average calculated average experimental shifts.

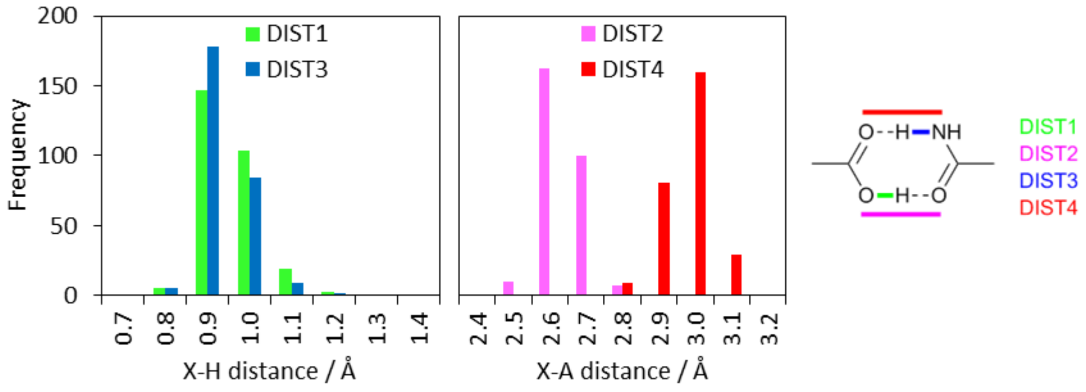

Figure S1 Histogram showing the distribution of X–H and X–A bond distances in COOH-CONH dimers.

IMPORTANT: this document contains embedded data - to preserve data integrity, please ensure where possible that the IUCr Word tools (available from http://journals.iucr.org/services/docxtemplate/) are installed when editing this document.

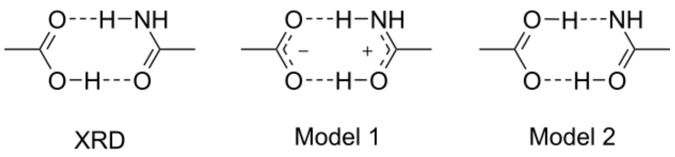

Figure S2 Schematic of the limiting structures of the different hydrogen bonding models investigated by DFT calculations.

The histograms shown in Fig. S1 were calculated by in a search of the CSD for the COOH-CONH fragments shown on the right, and the following constraints: 3D coordinates determined, not disordered, no powder structures and only organics. A total of 237 structures were found to contain at least one of these dimers.

Analogous searches for the fragments corresponding to the limiting forms of “Model 1” and “Model 2”, see Figure S2, yielded three structures (Jones *et al.*, 2012, Rybarczyk-Pirek, 2012, Eberlin *et al.*, 2013) containing Model 1 and none for Model 2.

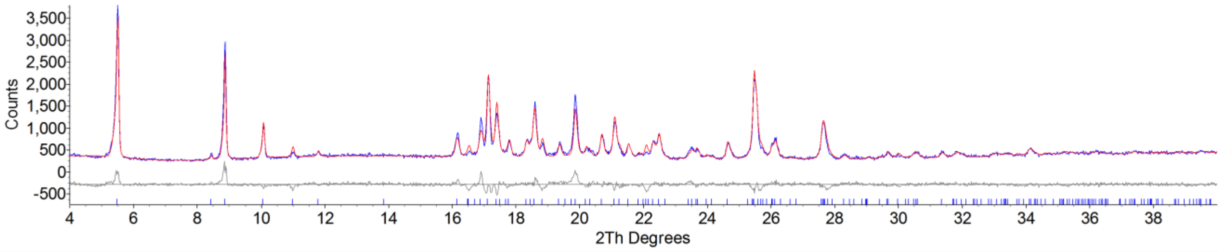

Figure S3 Rietveld fit of the XRPD pattern of the NPX-PA cocrystal; observed data are shown in blue, calculated pattern in red, difference curve in grey.

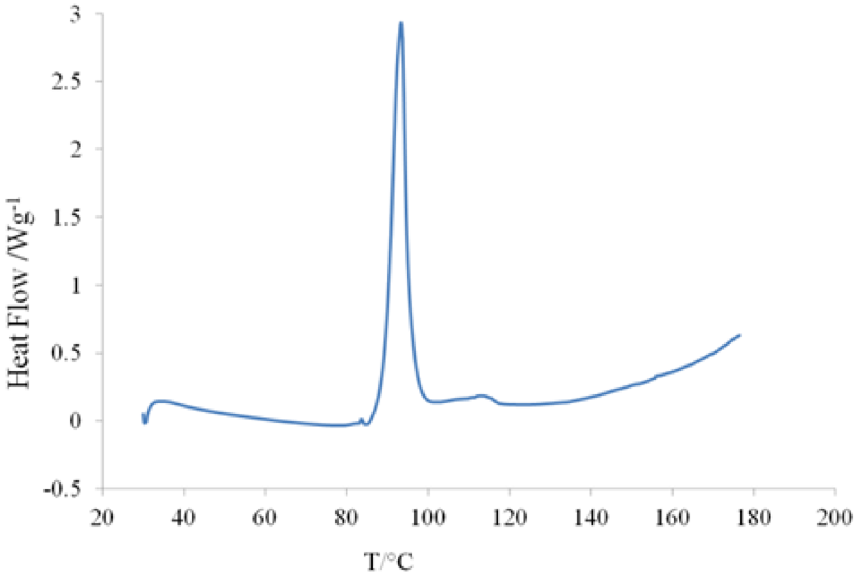

Figure S4 DSC trace of the NPX-PA cocrystal.

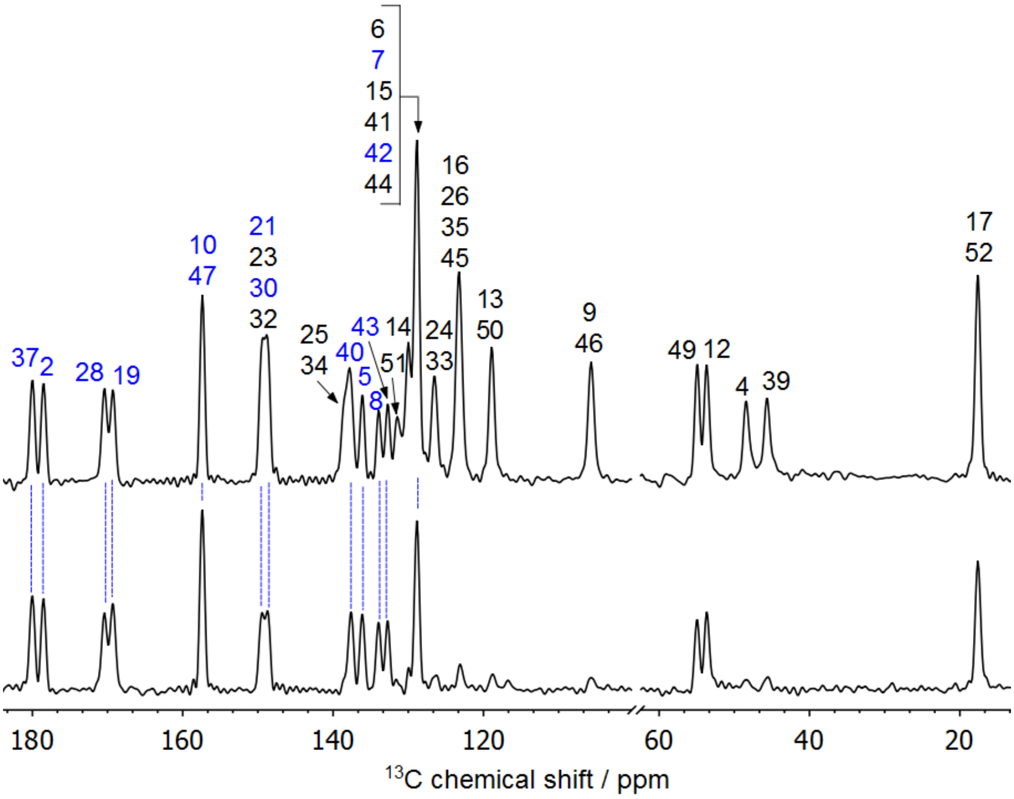

Figure S5 ^{13}C CP/TOSS (top) and CP/TOSS-NQS spectra of NPX-PA at a ^{13}C frequency of 125.7 MHz acquired with 5 kHz MAS. Assignments are given using the labelling scheme given in the main text, with quaternary carbon labels in blue.

^{13}C CP/MAS measurements with total sideband suppression were recorded on a 4 mm probe (rotor o.d.) with 8 kHz MAS, a recycle delay of 5 seconds and a contact time of 2.5 ms. Non-quaternary carbon peaks were suppressed by the addition of a dipolar dephasing delay of 80 µs. The assignment at 130 ppm is a little ambiguous due to the overlap of 8 signals, but C14 and C51 are assigned to 130 ppm and 131.4 ppm respectively from the lack of correlation of these peaks with any low shift protons (less than 4 ppm) in the HETCOR spectrum acquired with a long contact time (Fig. S5). These carbon sites are the only sites in the 130 ppm region that are further than 3 Å away from low shift protons. The order of the pairs of peaks was assigned using the CASTEP-calculated ordering. Other assignments were straight forward. Fig. S5 shows the ^{1}H – ^{13}C HETCOR spectrum acquired with a short contact time of 100 µs so only correlations between directly bonded atoms are visible. Fig. S5 also reveals differences in ^{1}H chemical shift between the two crystallographically unique dimers A and B: C4/C39 correlate to H41/H391, which differ in chemical shift by less than 0.5 ppm; C23/C32 correlate to H231/H321, which differ in chemical shift by roughly 1.5 ppm. This is consistent with the packing arrangement of the NPX and PA molecules: H41/H391 are in near identical environments in the two dimers but H231 is packed close to O1 unlike H321 which is near the C52 methyl group.

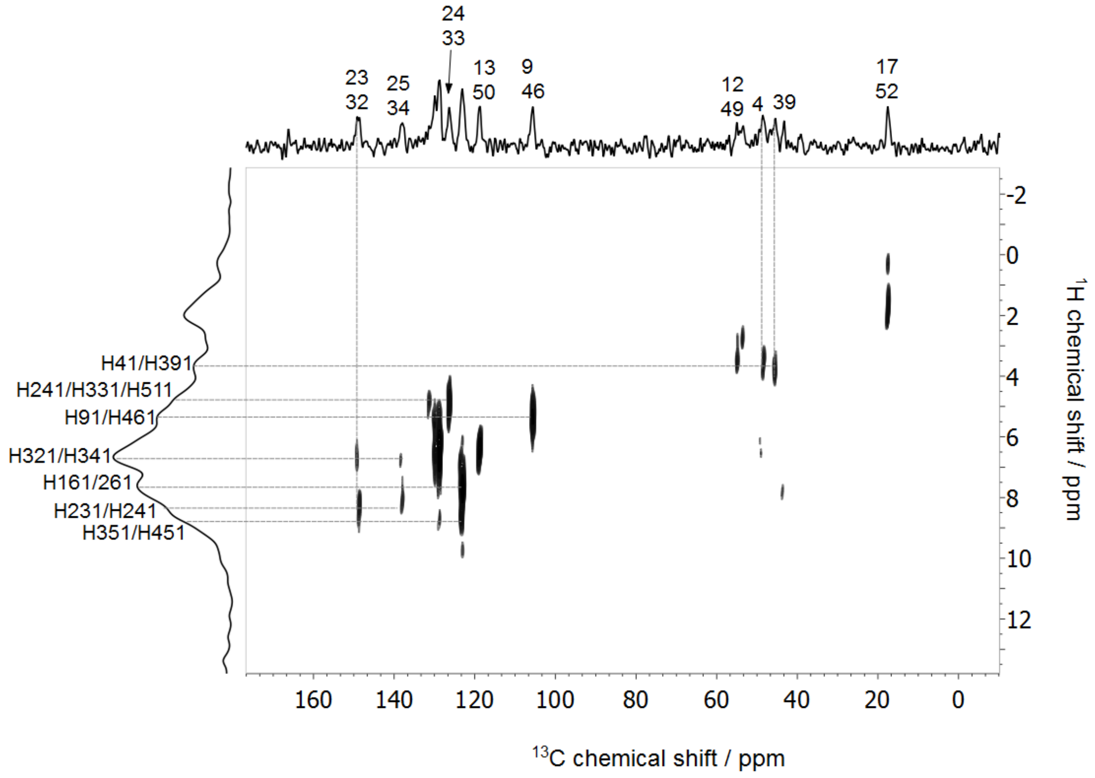

Figure S6 ^{1}H - ^{13}C HETCOR spectrum of NPX-PA at 10 kHz MAS with a 2 second recycle delay and a contact time of 100 µs.

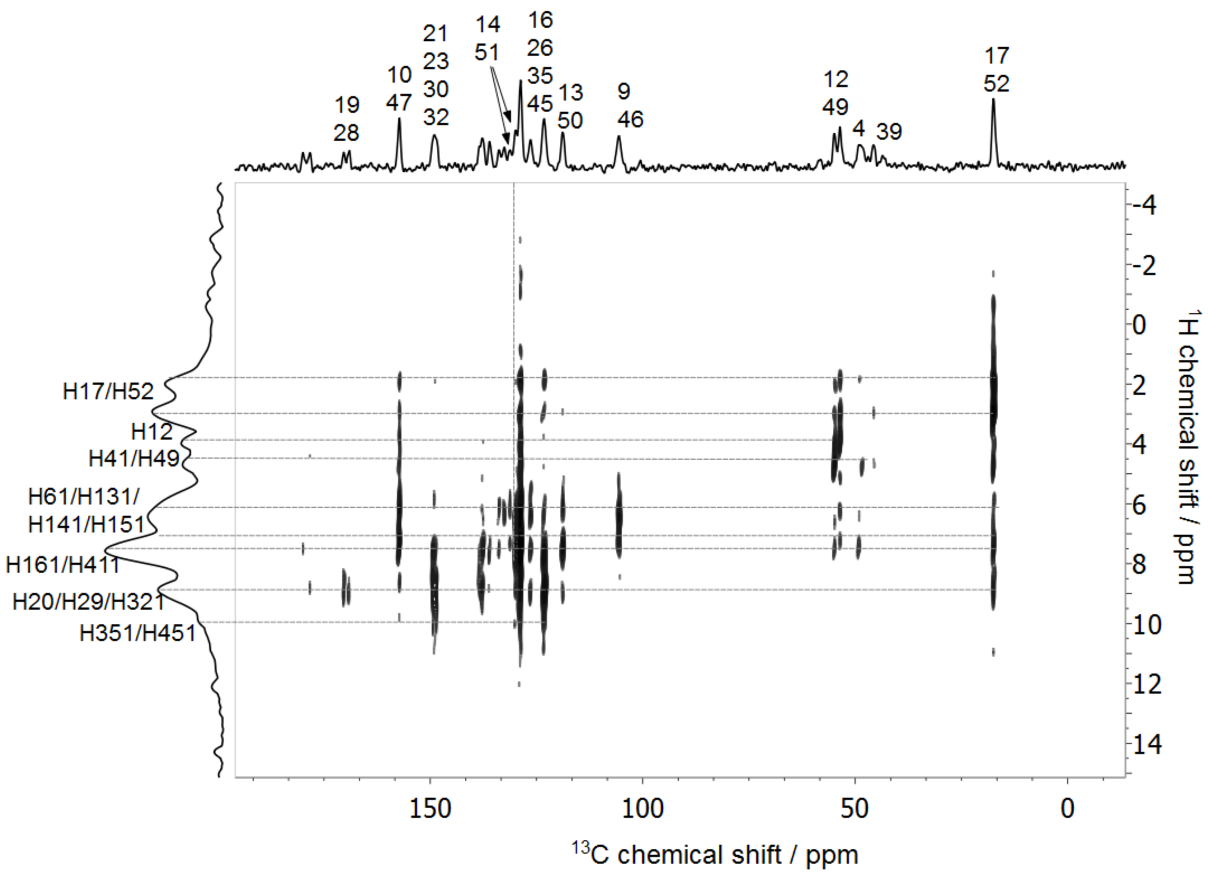

Figure S7 ^{1}H - ^{13}C HETCOR spectrum of NPX-PA at 10 kHz MAS with a 2 second recycle delay and a contact time of 1 ms.

Table S3 All carbon chemical shifts and assignments, along with predicted shieldings and the associated .magres labelling.

Magres Numbering	CIF label	Experimental shift[a] / ppm	Notes on assignment[b,c]
1	C2	178.5	C, Q
2	C4	48.6	C, S
3	C5	136.1	C, Q
4	C6	128.9	C?, S?, L? C6-H41 2.56 Å
5	C7	128.9	C?, Q
6	C8	134	C, Q
7	C9	105.7	C, S?
8	C10	157.4	C, L C10-H91 2.16 Å, C10-H131 2.16 Å
9	C12	53.7	C, S
10	C13	118.9	C, S?

11	C14	130	C?, S?, L?
12	C15	128.9	C?, S?, L? C15-H52 2.37 Å
13	C16	123.3	C, S?
14	C17	17.5	C, S
15	C19	169.4	C, Q
16	C21	148.9	C, Q
17	C26	123.3	C, S?
18	C25	138.3	C
19	C24	126.5	C, S?
20	C23	149.3	C, S?
21	C28	170.4	C, Q
22	C30	148.9	C, Q
23	C35	123.3	C, S?
24	C34	138.3	C
25	C33	126.5	C, S?
26	C32	149.3	C, S?
27	C37	180	C, Q
28	C39	45.7	C, S
29	C40	137.6	C, Q
30	C41	128.9	C?, S?, L? C41-H391 2.30 Å
31	C42	128.9	C?, Q
32	C43	132.8	C, Q
33	C44	128.9	C?, S?
34	C45	123.3	C, S?
35	C46	105.7	C, S?
36	C47	157.4	C, L C47-H461 2.16 Å, C47-H501 2.16 Å
37	C49	55	C, S
38	C50	118.9	C, S?
39	C51	131.4	C?, S?, L?
40	C52	17.5	C, S

[a] From CPTOSS spectrum acquired at a ^{13}C frequency of 125.67 MHz.

[b] Symbols used to indicate the basis of assignment: C = CASTEP-calculated ^{13}C shielding, Q = ^{13}C peak in non-quaternary suppression spectrum, S = cross peaks in HETCOR experiment with a short contact time, L = cross peaks in HETCOR experiment with a long contact time, ? = evidence is suggestive rather than definitive.

[c] Distances to selected non-bonded hydrogen atoms are given up to 2.70 Å.

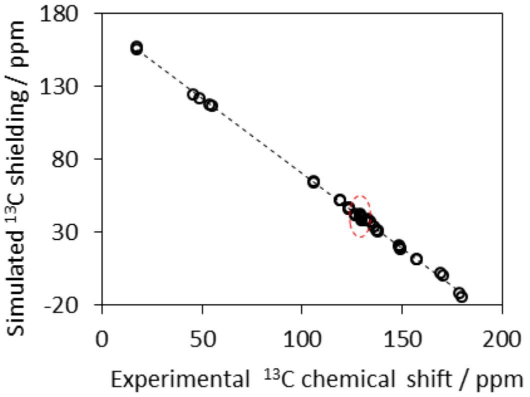

Figure S8 Correlation between calculated ^{13}C shielding values and experimental chemical shifts for NPX-PA. The dashed line is a linear regression highlighting the correlation. The red circle highlights the region where the assignment is ambiguous.

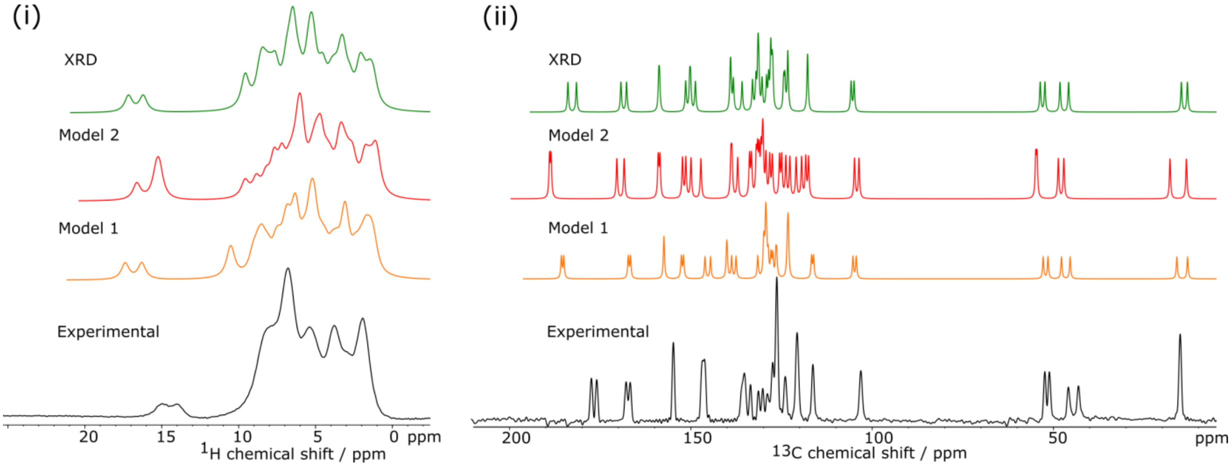

Figure S9 Comparison of the experimental ^{1}H spectrum (i) and ^{13}C spectrum (ii) of NPX-PA and the CASTEP-calculated spectra of NPX-PA from the structure refined from XRD data (green), Model 1 (orange) and Model 2 (red). The calculated spectra were referenced as described previously and modelled with 300 Hz and 50 Hz Lorentzian line broadening for ^{1}H and ^{13}C respectively. The predicted ^{1}H spectra for Model 1 and Model 2 poorly match the experimental ^{1}H spectrum as the amide protons H202/H291 are predicted to give rise to peaks at 11 ppm in Model 1 and 14 ppm in Model 2 that are not present experimentally.

The ^{1}H – ^{1}H double quantum-single quantum (DQ/SQ) spectrum was recorded using the back-to-back (BABA) sequence with 8 rotor cycles in the recoupling period and an evolution time of 1 μs. 128 t_1

increments were acquired with 16 transients per increment, which were co-added using the States-TPPI method. 200 Hz Gaussian line broadening was applied prior to FT. Simulated DQSQ spectra were produced using MagresView (Sturniolo *et al.*, 2016) and the point sizes were determined by the magnitude of the dipolar coupling, with a cut-off of 3.5 Å. The three simulated spectra were overlaid and then scaled as one to overlay with the experimental spectrum by eye.

The correlation peaks were assigned after measuring the ^{1}H – ^{1}H proximities in the geometry optimised structure, see Table S4. The CASTEP-calculated DQ/SQ correlations to H31 and H361 for the XRD-refined structure are shown in blue. These predicted DQ/SQ correlations are compatible with the experimental spectrum. It is noted that two strong correlations between H31 and H41 and H251 are not visible experimentally despite the distances being relatively small at 2.56 – 2.57 Å. This may be due to dipolar truncation, where strong dipolar coupling between two protons effectively swamps the weaker couplings to more distant neighbouring protons leading to potentially misleading correlation intensities (Bayro *et al.*, 2009, Hodgkinson & Emsley, 1999). Neither Model 1 nor 2 result in a simulated DQ/SQ spectrum that is compatible with the experimental spectrum. In particular the strong correlation to H361 is predicted in Model 1 to be much closer in DQ frequency to the strongest H31 correlation than is observed experimentally. There is also no correlation predicted for H361 at a DQ frequency less than 2 ppm, but the H361/H391 correlation is clearly visible experimentally.

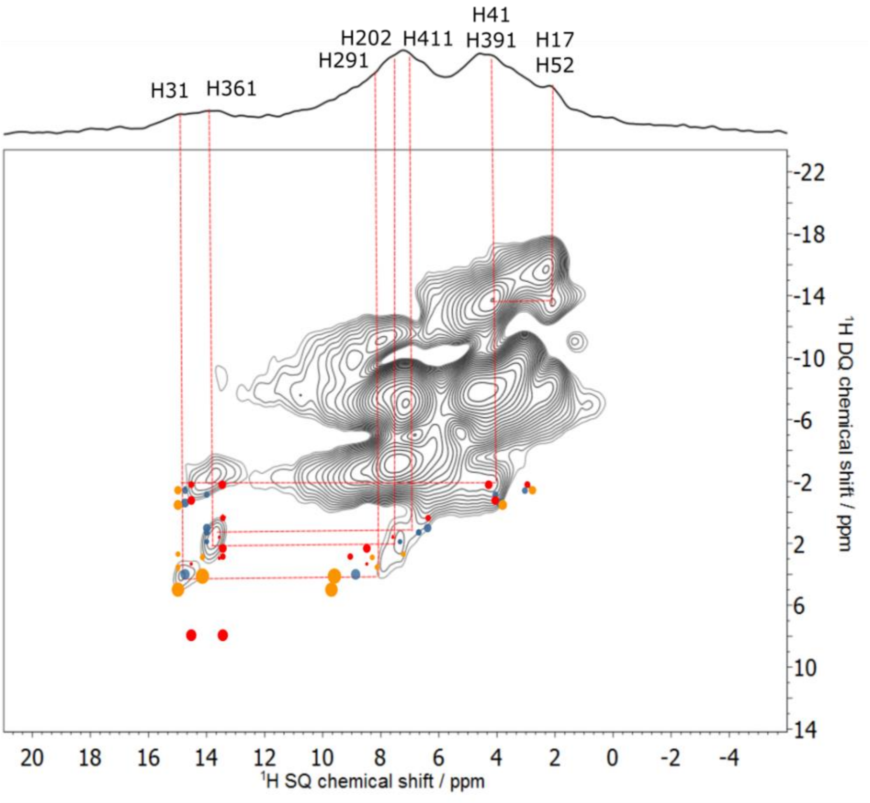

Figure S10 ^{1}H DQ/SQ SS-NMR spectrum of NPX-PA acquired at 60 kHz MAS. The labelled trace is a skyline projection. The CASTEP-calculated ^{1}H – ^{1}H correlations for the carboxyl protons from the optimised XRD structure are shown in blue and the CASTEP-calculated correlations for Model 1 are shown in orange and Model 2 in red. Larger dots represent stronger correlations.

Table S4 Selected ^{1}H – ^{1}H distances up to 3.0 Å measured in the optimised structures.

Correlation	XRD structure [a] / Å	Model 1 / Å	Model 2 [b] / Å
H31 – H291	2.39	2.29	2.21
H361 – H202	2.42	2.23	2.18
H31 – H41	2.56	2.64	2.53
H31 – H251	2.57	2.75	2.81
H361 – H411	2.59	2.72	2.68
H31 – H173	2.80	2.82	2.79
H361 – H391	2.84	2.69	2.68
H361 – H341	2.85	> 3.00	> 3.00
H31 – H351	2.89	> 3.00	> 3.00
H31 – H261	> 3.00	2.62	2.72
H361 – H351	> 3.00	2.96	> 3.00

H17/H52 – H41/391[c]	2.68	2.68	2.68

[a] All atom positions optimized.

[b] Only hydrogen atom positions optimised.

[c] Averaged over H171/2/3 and H41/2/3.

References

Bayro, M. J., Huber, M., Ramachandran, R., Davenport, T. C., Meier, B. H., Ernst, M. & Griffin, R. G. (2009). *J. Chem. Phys.*, **130**, 114506-114508.

Eberlin, A. R., Eddleston, M. D. & Frampton, C. S. (2013). *Acta Cryst.*, **C69**, 1260-1266.

Hodgkinson, P. & Emsley, L. (1999). *J. Magn. Reson.*, **139**, 46-59.

Jones, A. O., Lemee-Cailleau, M. H., Martins, D. M., McIntyre, G. J., Oswald, I. D., Pulham, C. R., Spanswick, C. K., Thomas, L. H. & Wilson, C. C. (2012). *Phys. Chem. Chem. Phys.*, **14**, 13273-13283.

Rybarczyk-Pirek, A. J. (2012). *Struct. Chem.*, **23**, 1739-1749.

Sturniolo, S., Green, T. F. G., Hanson, R. M., Zilka, M., Refson, K., Hodgkinson, P., Brown, S. P. & Yates, J. R. (2016). *Solid State Nucl. Magn. Reson.* 10.1016/j.ssnmr.2016.05.004.
